# GWAS and polygenic risk score of severe COVID-19 in Eastern Europe

**DOI:** 10.3389/fmed.2024.1409714

**Published:** 2024-09-19

**Authors:** Elena Kovalenko, Layal Shaheen, Ekaterina Vergasova, Alexey Kamelin, Valerya Rubinova, Dmitry Kharitonov, Anna Kim, Nikolay Plotnikov, Artem Elmuratov, Natalia Borovkova, Maya Storozheva, Sergey Solonin, Irina Gilyazova, Petr Mironov, Elza Khusnutdinova, Sergey Petrikov, Anna Ilinskaya, Valery Ilinsky, Alexander Rakitko

**Affiliations:** ^1^Genotek Ltd., Moscow, Russia; ^2^Genetic Technologies Ltd., Yerevan, Armenia; ^3^N.V. Sklifosovsky Research Institute for Emergency Medicine of Moscow Healthcare Department, Moscow, Russia; ^4^Institute of Biochemistry and Genetics, Ufa Federal Research Centre, Russian Academy of Sciences, Ufa, Russia; ^5^Bashkir State Medical University, Ufa, Russia; ^6^Eligens SIA, Mārupe, Latvia; ^7^Laboratory of Bioinformatics, Faculty of Computer Science, HSE University, Moscow, Russia

**Keywords:** COVID-19, GWAS, polygenic risk score, severe COVID-19, *LZTFL1* gene

## Abstract

**Background:**

COVID-19 disease has infected more than 772 million people, leading to 7 million deaths. Although the severe course of COVID-19 can be prevented using appropriate treatments, effective interventions require a thorough research of the genetic factors involved in its pathogenesis.

**Methods:**

We conducted a genome-wide association study (GWAS) on 7,124 individuals (comprising 6,400 controls who had mild to moderate COVID-19 and 724 cases with severe COVID-19). The inclusion criteria were acute respiratory distress syndrome (ARDS), acute respiratory failure (ARF) requiring respiratory support, or CT scans indicative of severe COVID-19 infection without any competing diseases. We also developed a polygenic risk score (PRS) model to identify individuals at high risk.

**Results:**

We identified two genome-wide significant loci (*P*-value <5 × 10^−8^) and one locus with approximately genome-wide significance (*P*-value = 5.92 × 10^−8^-6.15 × 10^−8^). The most genome-wide significant variants were located in the *leucine zipper transcription factor like 1* (*LZTFL1*) gene, which has been highlighted in several previous GWAS studies. Our PRS model results indicated that individuals in the top 10% group of the PRS had twice the risk of severe course of the disease compared to those at median risk [odds ratio = 2.18 (1.66, 2.86), *P*-value = 8.9 × 10^−9^].

**Conclusion:**

We conducted one of the largest studies to date on the genetics of severe COVID-19 in an Eastern European cohort. Our results are consistent with previous research and will guide further epidemiologic studies on host genetics, as well as for the development of targeted treatments.

## Background

Coronavirus infection, or COVID-19, caused by the SARS-CoV-2 virus, has resulted in one of the largest pandemics in human history (1). Epidemiological data on this disease emphasizes the remarkable heterogeneity in the course of the disease, ranging from completely asymptomatic cases to ICU hospitalizations with ventilator support and even fatal outcomes (2–5).

COVID-19 is associated with several comorbidities and non-genetic risk factors that increase the likelihood of developing the disease and experiencing severe progression, including respiratory failure. These risk factors include older age, male gender, and certain medical conditions such as cardiovascular disease, diabetes, obesity, chronic respiratory and kidney diseases, immunodeficiency, and neurological disorders (6–10).

The observed variability in susceptibility to and course of coronavirus infection suggests that both nongenetic risk factors and genetic variants may contribute to the clinical heterogeneity of COVID-19 in the population. Therefore, the COVID-19 Host Genetic Initiative (HGI) conducted a large study on a multiethnic sample of more than 49,500 COVID-19 patients from 46 studies in 19 countries (11). The study identified 13 genome-wide significant loci that are associated with SARS-CoV-2 infection or severe manifestations of COVID-19. Along with other genome-wide association studies (GWAS), several key genetic variants associated with severe COVID-19 outcomes were identified, which included those related to the function of the SARS-CoV-2 receptor (ACE2, ABO, TMPRSS2, and SLC6A20) (12–15) and immune response to the virus (HLA-region) (16, 17).

As epidemiological data and associated genetic variants for COVID-19 disease continue to accumulate, the combined assessment of disease severity for clinical implications remains an area of ongoing research. It is hypothesized that a combination of different gene variants may determine the severity of COVID-19 disease course. In other words, the complex polymorphic pathogenesis of coronavirus infection suggests a polygenic architecture.

Horowitz et al. (13) used the data from the COVID-19 HGI to establish a polygenic risk score (PRS) model. They demonstrated that individuals in the top 10% of the COVID-19 PRS among Europeans (*n* = 44.958) had a 1.38-fold increased risk of hospitalization and 1.58-fold increased risk of severe disease. Similarly, Farooqi et al. (18) reported a 1.57-fold higher risk of severe COVID-19 in high-risk patients compared to low-risk individuals. Another study by Crossfield et al. (19) involving 9,560 UK Biobank participants showed an adjusted odds ratio (OR) of 1.32, [95% confidence interval (CI): 1.11–1.58] for the highest PRS quintile compared with the lowest one. Nostaeva et al. (20) applied the same HGI statistics to a small cohort of Russian patients (1,085 participants, 347 individuals with severe COVID-19, and 738 with moderate or without disease) low-pass whole genome sequencing (LP-WGS). They found that more than one million genetic variants can be included in calculating polygenic scores to stratify patients by the risk of severe COVID-19. Individuals in the top 10% of the PRS distribution had over a two-fold increased risk of severe COVID-19 (odds ratio, OR: 2.2; 95% CI: 1.3–3.3, *P*-value = 0.0001).

Since coronavirus infection can develop rapidly in just over a week, identifying individuals at risk of developing severe COVID-19 through genetic variants may help identify targeting agents for investigating appropriate therapeutic interventions. Despite large-scale vaccination programs, optimal treatment selection remains a topical challenge. Therefore, the generation of a PRS model and patient profiling based on the risk of severe COVID-19 course can serve as a valuable tool for the healthcare industry.

In this research, we investigate the genetic factors that contribute to the severity of COVID-19 within the Eastern European population, which is underrepresented in many studies. By utilizing GWAS, we aim to identify genetic variants associated with COVID-19 and explore how these variants differ from those found in other populations. Additionally, we develop a PRS model to identify individuals at a high risk of experiencing severe COVID-19 outcomes.

## Materials and methods

### Study cohort

We analyzed the genetic data of 787 individuals, with 691 from the N.V. Sklifosovsky Research Institute for Emergency Medicine and 96 from the Ufa Federal Research Center of the Russian Academy of Sciences (UFRC RAS). Biomaterials, specifically blood or saliva, were collected from individuals who had a history of severe COVID-19.

Patients were classified as having an extremely severe course of COVID-19 if they met at least one of the following inclusion criteria: acute respiratory distress syndrome (ARDS); acute respiratory failure (ARF) requiring respiratory support, which could include high-flow non-invasive or invasive ventilation; lung changes on CT scans indicative of viral damage, such as significant or subtotal lesion volume (CT grade 4); or a clinical presentation consistent with ARDS. In addition, two mandatory criteria had to be met: a confirmed COVID-19 infection with the virus identified (ICD-10 code U07.1) and the absence of other diseases that could potentially worsen the patient's condition, such as acute myocardial infarction, exacerbation of bronchial asthma or chronic obstructive pulmonary disease (COPD), or decompensation of chronic heart failure.

This research was approved by the Genotek Ethics Committee (protocol No15 “GWAS of severe COVID-19 in the Russian population”) and performed in accordance with the Declaration of Helsinki. The individuals who were included in our analysis provided informed consent for their data to be used for research purposes and responded to an online questionnaire.

Genotek customer data were added to the study cohort for further analysis as controls (*N* = 6,400). The participants of the study were selected based on responses to a questionnaire, adhering to the following criteria: consent was provided for the use of anonymized data in scientific research and individuals aged 40 years or above should self-report a COVID-19 diagnosis, confirmed by antibody tests, PCR tests, CT scans, or a physician's diagnosis. These individuals may have experienced mild symptoms such as general fatigue, cough, and loss of smell or taste, along with other non-severe symptoms, without requiring hospitalization.

The number of cases was predetermined as we have obtained their data from hospitals. Although we had the option to adjust the number of cases by modifying the age threshold, we decided to maintain a ratio of ~10 controls per case to enhance the statistical power of our study. The rationale for selecting this sample size has been discussed in a study by Katki et al. (21).

### Genotyping

DNA extraction and genotyping were performed on saliva samples that were genotyped on Illumina Infinium Global Screening Array v.3 microarrays [~650,000 single nucleotide polymorphisms (SNPs)]. All samples in the cohort were processed in batches with 192–768 samples per batch using the Genotek microarray data processing pipeline. The pipeline involves variant detection based on iaap-cli 1.1.0 and bcftools +gtc2vcf plugin 1.11, followed by subsequent filtering and analysis. GenomeStudio software (Illumina, San Diego, CA) and manually created cluster files were used to cluster the raw signals and call the genotypes. SNPs with a call rate of < 0.9 within the batch were removed.

### Quality control and data preparation

Two-stage sequential filtering was performed based on the number of variants with undetermined genotypes. First, we filtered out the genetic variants with undetermined genotypes in more than 20% of the samples because the quality of detection of these variants was likely to be low, potentially leading to inaccurate conclusions in subsequent stages of the analysis. Subsequently, samples with undetermined genotypes in more than 20% of positions were excluded because the quality of collection, preparation, or analysis of these samples was likely to be low, which may lead to inaccurate conclusions in subsequent stages of the analysis. Following this step, filtering was repeated for positions and samples with a threshold of 2%.

After filtering for variant and sample quality, heterozygosity analysis was performed. The samples with abnormal heterozygosity were filtered using PLINK 1.9. We excluded the samples in which the observed heterozygosity deviated by more than 3 standard deviations from the mean. Heterozygosity estimation was performed on the cohort after filtering for genetic variants in linkage disequilibrium, using a search window of 50 SNPs, with five SNPs to shift the window at the end of each step, and an *r*^2^ value of SNPs < 0.2.

Subsequently, the human genotype was determined at positions not represented on the microarray by applying linkage disequilibrium (LD). This procedure was performed using the Beagle 5.1 program (22) using two reference panels: 1000 Genomes (23) and the Haplotype Reference Consortium (23, 24). Only those positions that achieved a high-quality metric for defining human genotypes with DR2 < 0.7 were used in further analysis. Multi-allelic substitutions were excluded from further analysis.

In the following step, the positions on sex chromosomes and mitochondrial DNA (mtDNA) were excluded. In addition, positions that violated the Hardy–Weinberg equilibrium were filtered out; specifically, we removed the positions with a significant difference between the observed genotype frequencies and the expected frequencies according to the Hardy–Weinberg test (*P*-value < 1 × 10^−5^). Finally, the positions with a low minor allele frequency (MAF < 0.05) were excluded from further analysis.

Identification of close relatives within the study cohort was performed using the PRIMUS (Rapid Reconstruction of Pedigrees from Genome-wide Estimates of Identity by Descent) program (25). Pairs with a PI_HAT score of >0.15 were considered as related. The cohort was filtered to ensure that it contained no pairs of relatives.

### Genome-wide association study (GWAS) and heritability

We performed population stratification and filtered outliers before conducting the GWAS analysis. Initially, the principal component analysis (PCA) algorithm (MultiDimensional Scaling) was applied for dimensionality reduction. Positions filtered by non-equilibrium coupling were used, considering a search window of 50 SNPs, with five SNPs to shift the window at the end of each step and an *r*^2^ value of SNPs < 0.2). Based on the values of the first and second components, clustering was performed using the DBSCAN algorithm (26). The final PCA plot is shown in Supplementary Figure S1. After selecting the largest cluster, outliers and samples not from this cluster were excluded. The top 20 components were subsequently used as covariates to account for population stratification.

The GWAS analysis was performed using the PLINK 2 program. A logistic regression model was used, and 20 components of PCA and gender were included as covariates.

The statistical package ldsc (https://github.com/bulik/ldsc) was used to estimate SNP heritability.

### Polygenic risk score

To construct the PRS model, summary statistics from the International Consortium COVID-19 Host Genetics Initiative (27) were employed (A2 phenotype, ALL_leave_23andme cohort, release 7). Variants with complementary alleles and variants with repeated rs_id were removed during data preprocessing. The PRS was trained using the LDPred2 tool (28) on the data from the entire cohort of 7,124 individuals. The scores were adjusted for sex and the first 20 components of PCA coordinates.

## Results

To account for population stratification, we constructed a PCA plot with two principal components (Supplementary Figure S1) and performed clustering to detect minor populations and outliers. The largest cluster (number 1) was selected for further analysis and all other samples were considered outliers.

We performed a GWAS analysis on 7,124 individuals (with 53% being female individuals) from the selected cluster. Among them, 6,400 individuals were controls (i.e., they were aged over 40 years and had recovered from COVID-19 without experiencing a severe outcome), and the remaining 724 patients were cases (i.e., they experienced a severe course of COVID-19). The mean age was 64.77 years for cases and 49.53 years for controls. The characteristics of the final cohort are summarized in Table 1.

**Table 1:** Characteristics of the study population.

**Type**	**Trait**	**Cohort (*N* = 7,124)**
Cases		724
	Age, years (SD)	64.774 (± 15.516)
	**Sex**	
	Female	381 (52.6%)
	Male	343 (47.4%)
Controls		6,400
	Age, years (SD)	49.528 (± 8.443)
	**Sex**	
	Female	3,398 (53.1%)
	Male	3,002 (46.9%)

**Figure 1:**
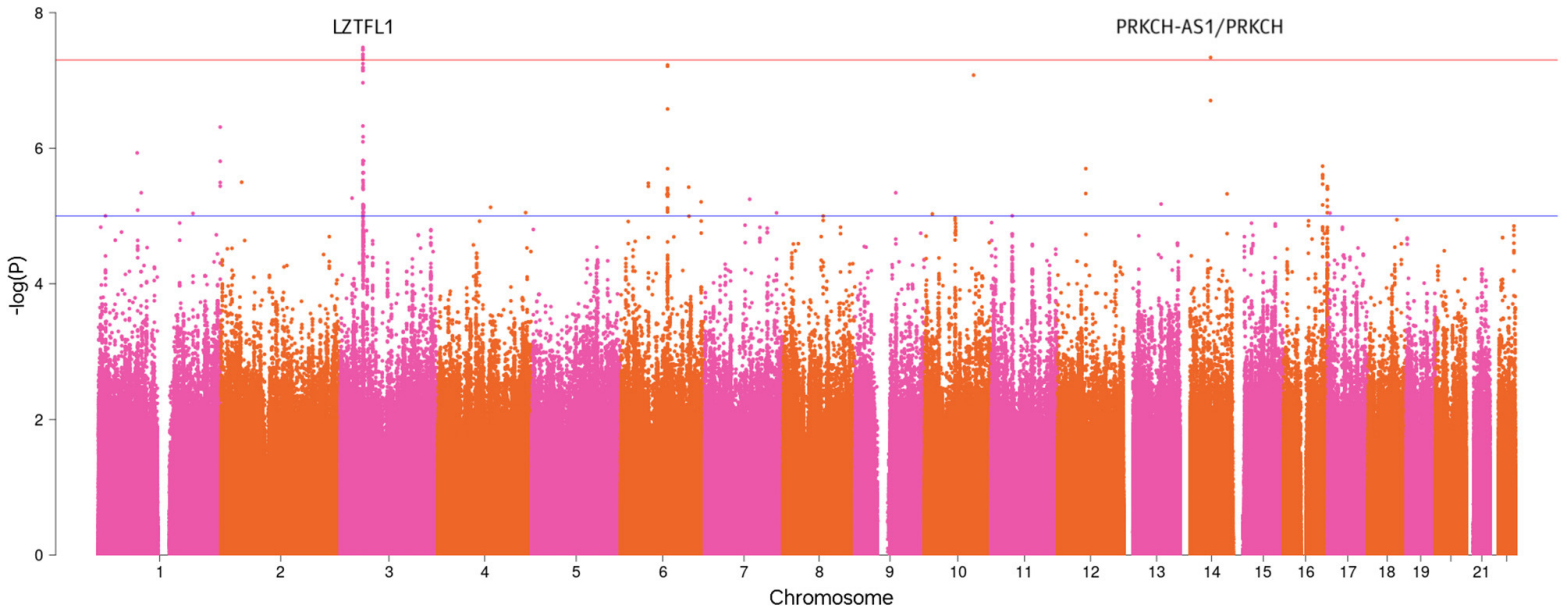
Manhattan plot with the GWAS results for the severe COVID-19 phenotype (*P*-value < 5 × 10^−8^).

The GWAS analysis revealed several genome-significant loci (*P*-value < 5 × 10^−8^), (Table 2). Figure 1 shows the Manhattan plot with the GWAS results.

**Table 2:** Top GWAS results (*P*-value <10^−8^).

**Gene**	**rsid**	**chr**	**pos_hg19**	**ref. allele**	**alt. allele**	**eff. allele**	**EAF**	**OR**	**lower _CI**	**upper _CI**	***P***
**LZTFL1**	**rs35624553**	**3**	**45867440**	**A**	**G**	**G**	**0.102**	**1.575**	**1.341**	**1.850**	**3.26E-08**
**intergenic**	**rs17713054**	**3**	**45859651**	**G**	**A**	**A**	**0.102**	**1.573**	**1.339**	**1.848**	**3.51E-08**
**intergenic**	**rs13078854**	**3**	**45861932**	**G**	**A**	**A**	**0.102**	**1.573**	**1.339**	**1.848**	**3.51E-08**
**intergenic**	**rs71325088**	**3**	**45862952**	**T**	**C**	**C**	**0.102**	**1.573**	**1.339**	**1.848**	**3.51E-08**
**LZTFL1**	**rs10490770**	**3**	**45864732**	**T**	**C**	**C**	**0.102**	**1.573**	**1.339**	**1.848**	**3.51E-08**
**LZTFL1**	**rs67959919**	**3**	**45871908**	**G**	**A**	**A**	**0.102**	**1.570**	**1.336**	**1.844**	**4.11E-08**
**intergenic**	**rs13071258**	**3**	**45843242**	**G**	**A**	**A**	**0.104**	**1.563**	**1.332**	**1.833**	**4.17E-08**
**intergenic**	**rs34668658**	**3**	**45844198**	**A**	**C**	**C**	**0.104**	**1.563**	**1.332**	**1.833**	**4.17E-08**
**intergenic**	**rs17763742**	**3**	**45846769**	**A**	**G**	**G**	**0.104**	**1.561**	**1.331**	**1.831**	**4.51E-08**
**PRKCH- AS1/ PRKCH**	**rs1989566**	**14**	**61777603**	**C**	**A**	**A**	**0.038**	**1.928**	**1.524**	**2.440**	**4.59E-08**
**LZTFL1**	**rs35652899**	**3**	**45908514**	**C**	**G**	**G**	**0.100**	**1.574**	**1.337**	**1.853**	**4.85E-08**
LZTFL1	rs13081482	3	45908116	A	T	T	0.100	1.571	1.334	1.849	5.66E-08
intergenic	rs447073	6	95228012	T	C	C	0.346	0.715	0.634	0.807	5.92E-08
intergenic	rs222542	6	95231062	A	G	G	0.346	0.715	0.634	0.808	6.15E-08
intergenic	rs222541	6	95231163	G	C	C	0.346	0.715	0.634	0.808	6.15E-08
LZTFL1	rs35508621	3	45880481	T	C	C	0.102	1.563	1.329	1.837	6.48E-08
LZTFL1	rs34288077	3	45888690	A	G	G	0.102	1.563	1.329	1.837	6.48E-08
LZTFL1	rs35044562	3	45909024	A	G	G	0.101	1.567	1.331	1.845	6.59E-08
intergenic	rs17763537	3	45843315	C	T	T	0.103	1.557	1.326	1.828	6.67E-08
LZTFL1	rs35081325	3	45889921	A	T	T	0.101	1.565	1.330	1.843	7.12E-08
LZTFL1	rs35731912	3	45889949	C	T	T	0.101	1.565	1.330	1.843	7.12E-08
LZTFL1	rs34326463	3	45899651	A	G	G	0.101	1.565	1.330	1.843	7.12E-08
LZTFL1	rs73064425	3	45901089	C	T	T	0.101	1.565	1.330	1.843	7.12E-08
CRTAC1	rs3793697	10	99638216	T	G	G	0.158	1.447	1.264	1.657	8.36E-08

Genome-wide significant results are indicated in bold.

Particularly, the most genome-wide significant loci were identified in the *leucine zipper transcription factor like 1* (*LZTFL1*) gene: rs35624553 (OR = 1.58, 95% CI: 1.34–1.85, *P*-value = 3.26 × 10^−8^) and rs10490770 (OR = 1.57, 95% CI: 0.082–1.34, *P*-value = 3.51 × 10^−8^). These findings were previously mentioned in the summary statistics of the COVID-19 HGI relevant to our phenotype. Three intergenic variants on chromosome 3 (rs17713054, rs13078854, and rs71325088) had the same association with severe forms of COVID-19 as observed with rs10490770.

**Figure 2:**
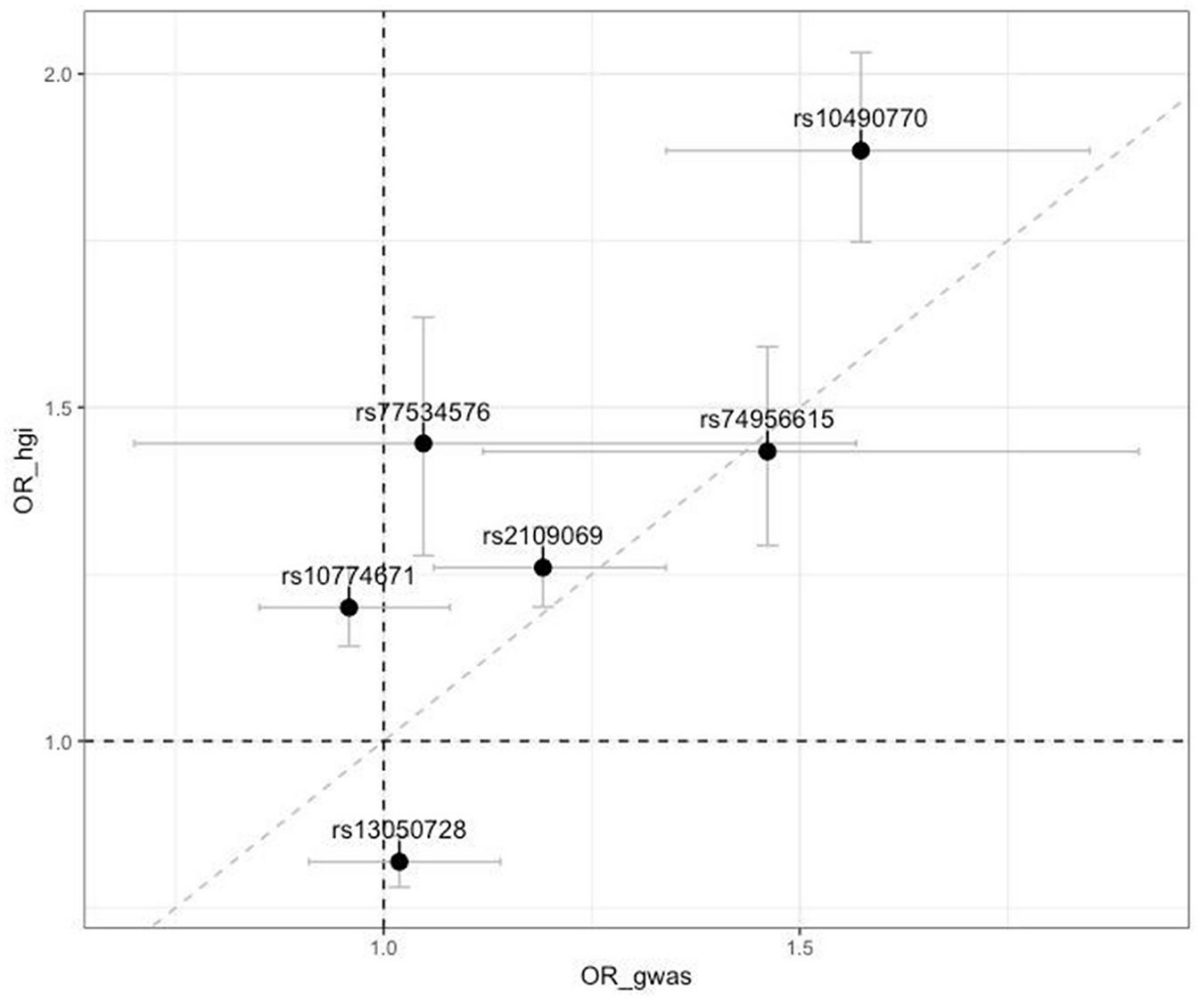
Scatter plot of comparison of odds ratios (OR) from the top associated SNPs from the COVID-19 Host Genetics Initiative with our results (OR_hgi indicates results from COVID-19 HGI GWAS and OR_gwas indicates results from the current study). Horizontal and vertical bars represent 95% confidence intervals.

Furthermore, we compared the effects of SNPs identified by the COVID-19 HGI (11), which are associated with critical illness (similar to our phenotype) and the current GWAS results. We could not compare the effect of rs912805253 as it is absent in hg19. The remaining SNPs exhibited poor approximation with HGI results; however, the intergenic variants highlighted in the HGI summary statistics (rs1819040 and rs74956615) demonstrated the effects that were comparable to our results (rs1819040: OR_hgi_ = 0.906, OR_genotek_ = 0.920, rs74956615: OR_hgi_ = 1.434, OR_genotek_ = 1.461). Figure 2 illustrates these results.

The heritability h2 coefficient was found to be 0.05 ± 0.0432.

**Figure 3:**
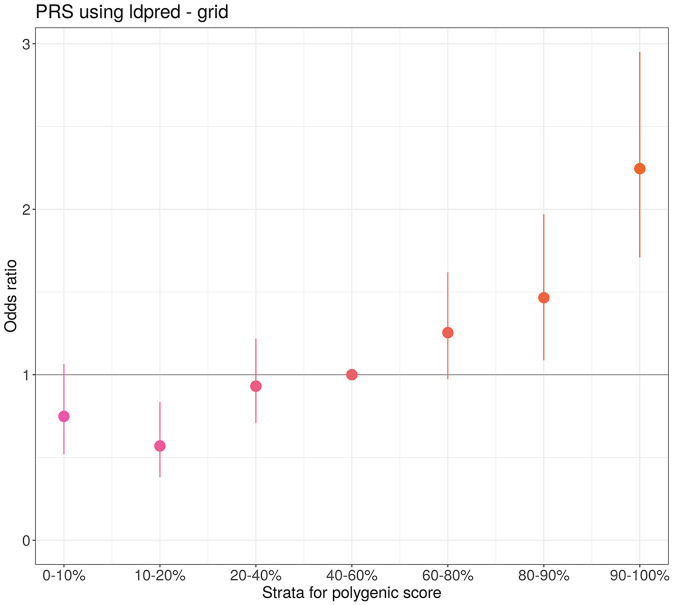
Quantile plot of the severe course of COVID-19 PRS developed by LDPred2. The odds ratio represents comparison of PRS odds from different quantiles with the reference quantile (40%−60%). The bars represent the standard deviation (SD).

Furthermore, we generated the PRS model to identify the group of individuals with a high risk of severe COVID-19 (Figure 3). In this group, the risk of experiencing a severe course of the disease is approximately twice that of the median risk [with OR = 2.25 (1.7, 2.95), *P*-value = 3.1 × 10^−9^]. The area under the curve (AUC) for the developed PRS is equal to 0.6 (0.58–0.62). The PRS was constructed using the grid model in LDPred2 and included 955,503 SNPs.

## Discussion

In this study, we identified several genome-wide significant loci associated with the severe forms of COVID-19. The loci include *LZTFL1* and *PRKCH-AS1/PRKCH* genes, intergenic variants on chromosome 3, and a novel loci with intergenic variants on chromosome 6 with genome-wide significance (*P*-value = 5.92 × 10^−8^ to 6.15 × 10^−8^). In addition, several genetic variants in the *LZTFL1* gene showed genome-wide significance.

The intron variant rs1989566, located within the *PRKCH-AS1/PRKCH* gene, was newly associated with the susceptibility and severity of COVID-19. The PKC family serves as a mediator for diverse signaling pathways and governs numerous crucial cellular functions, including proliferation, differentiation, and apoptosis. One of the PKC family members, protein kinase C eta protein (PKCη), which is encoded by the *PRKCH* gene, is a serine-threonine kinase. It is predominantly expressed in vascular endothelial cells and plays a role in the progression and exacerbation of atherosclerosis and subsequently stroke, as indicated by previous studies (29, 30).

Furthermore, the identified variant may potentially impact the expression of the *PRKCH-AS1/PRKCH* gene and subsequently disrupt endothelial function, which can cause an imbalance in hemostasis favoring a procoagulant state. This is characterized by impaired vasodilator release, increased release of vasoconstrictors, heightened microvasculature spastic reactions, increased leukocyte migration across the endothelium, and the initiation of localized inflammation. Prolonged exposure to factors that induce endothelial dysfunction can contribute to a pro-inflammatory and prothrombotic phenotype in endothelial cells (31). This exposure also results in a depletion of the pool of progenitor endothelial cells (32), ultimately limiting the capacity for restoring their normal phenotype and function.

In addition to regulating cell proliferation, differentiation, and cell death, PKCη is also expressed in the lung tissue, immune system, and proliferation pathways, for instance, in activating nuclear factor κB (NF-κB) signaling, which leads to anti-cancer drug resistance (33–35).

We confirmed the effect of rs10490770 on severe course of COVID-19 (OR = 1.57, *P*-value = 3.51 × 10^−8^) in the *LZTFL1* gene. Previous studies (11, 36) have shown that rs10490770 increases the risks of all-cause mortality (HR = 1.4), severe respiratory failure (OR, 2.1), venous thromboembolism (OR = 1.7), and hepatic injury (OR = 1.5). For elderly people, it significantly increases the risk of mortality or severe respiratory failure by more than a double (OR, 2.7).

The *LZTFL1* gene, expressed in the normal lung epithelium, is involved in protein transport to the cilia of the ciliated epithelium respiratory cells (37). The transcriptome analysis of lung biopsies from patients with COVID-19 showed the presence of signals associated with epithelial-mesenchymal transition of lung cells (EMT) or pulmonary fibrosis, which is regulated by *LZTFL1*, suggesting that this locus may serve as a potential therapeutic target (38). Other studies have also linked the *LZTFL1* gene at the 3p21.31 locus to COVID-19 infection (39, 40).

Several limitations could affect our GWAS results, including relatively small number of controls, low trait heritability (h2 coefficient), and high polygenicity. Previous research has established that age is a significant risk factor for severe COVID-19, with older individuals being at a higher risk. In our study, the patient cases were sourced from hospitals with an average age of 64.77 years, while controls were selected from the database of a genetic testing company, Genotek, with an average age of ~35 years. We did not use a population control, as is commonly done in many studies. Instead, we defined controls as individuals aged 40 years or above who reported having COVID-19 but did not experience a severe course of the disease. The age threshold was set at 40 years to balance the age distribution and the sizes of our case and control cohorts.

Our primary aim was to evaluate the predictive ability of the PRS of COVID-19 severity in the Eastern European cohort. Therefore, we performed the GWAS analysis on a cohort of 7,124 individuals and constructed a polygenic risk model. Stratification of individuals based on PRS quantiles revealed that the high-risk category (top 10%) had twice the risk of severe course of COVID-19 compared to the median risk group. These results support findings from previous European cohort studies demonstrating similar associations of the PRS with severe COVID-19 (13, 18–20). Before implementation, the PRS must be validated in independent cohorts. Additional prospective studies can be beneficial to assess the clinical utility of the PRS in a practical setting. The potential application of the PRS could involve stratifying all tested individuals into high- and low-risk groups. In our study, we demonstrated the predictive power of the developed PRS. The selection of a threshold for the PRS that delineates the high-risk group should be determined based on factors such as the anticipated increase in hospitalization risk, mortality rates, and economic considerations.

## Data Availability

For the Genotek dataset, the user agreement (available at https://www.genotek.ru) states that disclosure of individual-level genetic information and/or self-reported information to third parties for research purposes will not occur without explicit consent, and the consent was not obtained from the individuals. Due to the user agreement, the individual level cannot be made directly available, and the dataset could pose a threat to confidentiality. Data have to be accessed indirectly via Genotek Ltd, https://www.genotek.ru/. Data requests should be sent to Genotek Ltd at info@genotek.ru. The summary statistics of the severe course of COVID-19 GWAS based on the 7,124 Eastern-European participants is freely available at the NHGRI-EBI GWAS Catalog (https://www.ebi.ac.uk/gwas/home) with the accession number GCST90444397. PRS weights are available via the PGS catalog (https://www.pgscatalog.org), with the publication ID: PGP000666 and score IDs: PGS004938.
