# Supplementary material for: GWAS and polygenic risk score of severe COVID-19 in Eastern Europe
Source: Front Med (Lausanne). 2024 Sep 19;11:1409714. doi: 10.3389/fmed.2024.1409714 (PMC11446758; doi:10.3389/fmed.2024.1409714)
Supplement: Supplementary file 1 [file Data_Sheet_1.docx]

Supplementary Material


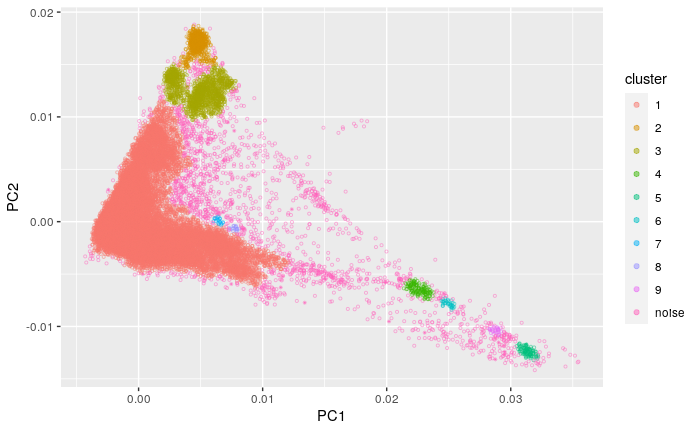


**Supplementary Figure 1.** PCA-plot of the study cohort. Dots are colored according to DBSCAN clustering.
